# Supplementary material for: Transcriptome Analysis Reveals Vimentin-Induced Disruption of Cell–Cell Associations Augments Breast Cancer Cell Migration
Source: Cells. 2022 Dec 13;11(24):4035. doi: 10.3390/cells11244035 (PMC9776984; doi:10.3390/cells11244035)
Supplement: Supplementary file 1 [file cells-11-04035-s001.zip › DEGs and enrichment analysis.zip!/DEGs and enrichment analysis/WT_VIMvsVector KEGG/DOWN/WT_VIMvsVector_DOWN.pathway_map.zip!/WT_VIMvsVector_DOWN.pathway_map/src/hsa04080.html]

hsa04080


- hsa:1081

- Down regulated genes

ENSG00000135346(-3.27212981463669)

- hsa:1081

- Down regulated genes

ENSG00000135346(-3.27212981463669)

- hsa:1081

- Down regulated genes

ENSG00000135346(-3.27212981463669)

- hsa:2898

- Down regulated genes

ENSG00000164418(-2.81140636790949)

- hsa:9568

- Down regulated genes

ENSG00000136928(-2.38971662645656)

- hsa:9340

- Down regulated genes

ENSG00000065325(-1.89002305043427)

- hsa:624

- Down regulated genes

ENSG00000168398(-1.85119834379304)

Close
